# Overexpression of AQP3 Modifies the Cell Cycle and the Proliferation Rate of Mammalian Cells in Culture

**DOI:** 10.1371/journal.pone.0137692

**Published:** 2015-09-14

**Authors:** Ana Galán-Cobo, Reposo Ramírez-Lorca, Ana Serna, Miriam Echevarría

**Affiliations:** 1 Instituto de Biomedicina de Sevilla (IBiS), Hospital Universitario Virgen del Rocío/CSIC/Universidad de Sevilla (Departamento de Fisiología Médica y Biofísica), Seville, Spain; 2 Centro de Investigación Biomédica en Red sobre Enfermedades Respiratorias (CIBERES), Instituto de Salud Carlos III, Madrid, Spain; Universidad de La Laguna, SPAIN

## Abstract

Abnormal AQP3 overexpression in tumor cells of different origins has been reported and a role for this enhanced AQP3 expression in cell proliferation and tumor processess has been indicated. To further understand the role AQP3 plays in cell proliferation we explore the effect that stable over expression of AQP3 produces over the proliferation rate and cell cycle of mammalian cells. The cell cycle was analyzed by flow cytometry with propidium iodide (PI) and the cell proliferation rate measured through cell counting and BrdU staining. Cells with overexpression of AQP3 (AQP3-o) showed higher proliferation rate and larger percentage of cells in phases S and G2/M, than wild type cells (wt). Evaluation of the cell response against arresting the cell cycle with Nocodazole showed that AQP3-o exhibited a less modified cell cycle pattern and lower Annexin V specific staining than wt, consistently with a higher resistance to apoptosis of AQP3-overexpressing cells. The cell volume and complexity were also larger in AQP3-o compared to wt cells. After transcriptomic analysis, RT-qPCR was performed to highlight key molecules implicated in cell proliferation which expression may be altered by overexpression of AQP3 and the comparative analysis between both type of cells showed significant changes in the expression of Zeb2, Jun, JunB, NF-kβ, Cxcl9, Cxcl10, TNF, and TNF receptors. We conclude that the role of AQP3 in cell proliferation seems to be connected to increments in the cell cycle turnover and changes in the expression levels of relevant genes for this process. Larger expression of AQP3 may confer to the cell a more tumor like phenotype and contributes to explain the presence of this protein in many different tumors.

## Introduction

Different key roles for AQPs have been associated with tumor biology including facilitation of cell migration, adhesion and cell proliferation. Although most works indicated that AQPs are overexpressed in the large variety of human tumors analyzed, reduced expression of these proteins have been demonstrated as well in some cases [1]. Enhanced expression of AQP3 was reported, among others, in colorectal carcinogenesis [2], human lung [3], gastric adenocarcinomas [4] and human skin squamous cell carcinomas [5–7]. AQP3 facilitates skin keratinocyte migration and proliferation [6], and deletion of this protein prevented skin tumor formation and retarded wound healing in an *in vitro* migration scratch assay in mice [4,5]. A widely accepted idea to explain the role of AQP3 in tumor cell proliferation allude to the fact that expression of this protein confers to the cell with a higher glycerol permeability and ATP content, which are required for a greater biosynthesis demand [8]. AQP5, an orthodox AQP strictly permeable to water and not to glycerol [9–11], has been also directly associated with cell proliferation [2,12–15], but oncogenic properties of AQP5 were related with activation of Ras, ERK and phosphorylation of retinoblastome (Rb), that will ultimately cause transcription of genes implicated with cell proliferation, growth and survival [15]. Overexpression of AQP5 was reported in colorectal carcinogenesis [2,15], non-small cell lung cancer [12], chronic myelogenous leukemia [13], and in human breast cancer [14]. In all those cases the oncogene role of AQP5 was more associated to phosphorylation and/or activation of signaling pathways for proliferation, than to the water transport capacity of the protein. Thus, whether or not the water and/or glycerol transporting functions of AQPs by itself would be necessary to increase cell proliferation remain still unclear.

Previously, we showed that stable overexpression of AQP1, 3 and 5 increases the stability of HIF-2α during chronic exposure to hypoxia [16,17]. The expression of many genes implicated in activities relevant for tumor growth, such as glucose uptake and metabolism, angiogenesis, cell proliferation and apoptosis are induced by HIF [18]. Hence the similar effect over HIF stability displayed by the three AQPs would suggest a common mechanism in this process [17].

More recently we demonstrated that inhibition of AQP3 with the gold-based compound, Auphen, strongly reduce the proliferation rate of cells that express AQP3 [19]. Cells treated with Auphen become arrested in the S-G2/M phases of the cell cycle denoting the possibility that the inhibition of AQP3´s permeability some how restrain progression of the cell cycle and thus lowering cell proliferation. Only few previous studies analyzed the connections between AQPs and cell cycle. Thus, it was indicated that AQP2 participates in the acceleration of cell proliferation *per se* in cells of the renal collecting duct, by increasing the rate of cell cycle progression [20,21]. More recently, in esophageal squamous cell carcinoma was indicated that AQP5 expression might affect the cell proliferation by affecting the expression of genes involved in cell cycle progression [22], and similar results were obtained in PC12 cells with stable overexpression of AQP1 where Western blot and Affymetrix assays confirmed changes in the expression of proteins and genes relevant for the cell cycle progression [23]. In the present work is shown that overexpression of AQP3 increases cell proliferation, probably by accelerating the cell cycle progression. Overexpression of AQP3 modifies the cell cycle pattern and the cell response to incubation with nocodazole. Large cell volume and complexity, as well as enhanced hydrogen peroxide permeability and altered expression of proteins necessary for progression of the cell cycle were confirmed; evidencing that the relationship between AQP3 and cancer goes beyond their water/or glycerol permeabilities.

## Materials and Methods

### Cell culture

#### PC12-wt

Cell line derived from rat adrenal gland pheochromocytoma previously used in our laboratory [24]. These cells were cultured in DMEM media with 10% horse serum, 5% FBS and 1% penicyline/streptomycine at 37°C and 10% CO_2_. The medium was changed every 2–3 days and 1:5 dilution passes were done every 5 days.

#### PC12-AQP3

PC12 cells stably transfected with AQP3 for overexpression of this protein. Protocols to obtain this cell line were described previously [16]. To culture these cells DMEM media was used with 10% horse serum, 5% FBS, 1% penicillin/streptomycin and 2mg/ml Geneticin and they were maintained at 37°C and 10% CO_2_. Medium was changed every 2–3 days. Dilution passes were done at 1:5 ratios every 5 days.

### Proliferation assays

#### Proliferation measurement by counting live cells in haemocytometer chamber after trypan blue staining

Cell lines were plated in 60 mm diameter dishes (Nunc, Thermo Fisher) at initial concentrations of 0.9 x 10^6^ cells for PC12-wt and PC12-AQP3 and after cultured during either: 0, 24, 48, 72 and 96 hours. Cells were trypsinized and resuspended in 1 mL of fresh medium, stained with trypan blue during 5 minutes and living cells counted using a haemocytometer chamber.

#### Proliferation rate measurement by BrdU incorporation analysis

Cells were plated at proper concentration on cover glasses pre-treated with poly-l-lysine (Sigma) diluted 1:10 in water. After 24 h, cells were incubated with 10 μM BrdU (Sigma—Aldrich) during 8 h and BrdU^+^ cells were count by immunofluorescence assay as indicated below.

#### Indirect immunofluorescence assay

Cells were fixed during 30 min in 95% acetic acid and 5% ethanol, washed three times during 5 min with PBS and incubated during 30 min in 2 M HCl. Cells were then neutralized with sodium-borate twice during 10 min and then blocked with 10% goat serum and 3% BSA in PBS, Incubation was made over night at 4°C with 1:300 Anti-BrdU anti-mouse antibody (G3/G4 Hybridoma Bank). After 24 h, cells were washed twice with PBS and then incubated with Alexa 488 anti-mouse (Invitrogen, Carlsbad, CA) 1:300 during 1 h at room temperature. Then, cells were washed three times during 5 min in PBS and incubated with DAPI 1:1,000 diluted in water during 10 min. Finally, cells were washed twice with PBS, mounted with DAKO (Invitrogen) and preserved at 4°C. Results were analyzed using a fluorescence microscope Olympus BX 71 with a refrigerated camera DP70. The number of BrdU^+^ cells was expressed as percentage and calculated relative to the total number of cells counted in the microscope field. Twenty fields were analyzed and averaged per each condition to give the final data shown.

### Cell cycle analysis

PC12 cells, both wild type and stably transfected with AQP3 for overexpression, were seeded in 60 mm dishes (Nunc, Thermo Fisher) at a density of 1.7x10^6^ cells/well. After 72 h of culturing, cells were harvested and cell suspensions were pelleted and washed twice with PBS. Cells were fixed with cold 70% ethanol for at least 24 h (4°C) and then centrifuged 5 minutes at 500 x g (room temperature). Cells were washed twice with FACS buffer and treated with 200 μg/ml RNase A (Sigma) for 1 hour (37°C). Then stained for DNA labeling with 40 μg/ml propidium iodide (PI) solution (Calbiochem) during 20 minutes (4°C) and transferred to flow cytometry tubes for cell cycle analysis in a BD LSR Fortessa flow cytometer (BD Biosciences). Cell cycle analysis was performed using the FACS Diva versión 6.3 (BD Biosciences).

#### G2/M synchronization

To block cell cycle progression at M phase, cells were cultured for 48 h in 60 mm dishes, and then nocodazole was added to the culture medium to final concentrations of 5, 7.5 and 10 μM (Sigma). After 24 h of exposure, cells were collected, washed and resuspended in FACS for PI staining and cell cycle analysis.

### Annexin apoptosis assays

For PC12-wt and PC12-AQP3 1×10^6^ cells were plated on 60mm dishes (Nunc) during 48 h. After this time cells were treated with 7.5 μM of nocodazole (Sigma) for 24 h. Following, cells were harvested and washed with FACS, and stained with the commercial kit Annexin-V-FITC apoptosis detection kit (Inmunostep) to analyze alive, early and late apoptotic and necrotic cells by flow cytometry using a BD LSR Fortessa flow cytometer (BD Biosciences).

### Cell size and complexity

The relative cell size and complexity of PC12-wt and PC12-AQP3 cell lines were evaluated by flow cytometry using FSC and SSC parameters in an LSRFortessa cytometer as indicated by the manufacturer (BD Biosciences).

### Western blotting

Cells were washed with cold PBS, collected by scraping in 1 ml of cold PBS and centrifuged at 300 ×g for 5 min at 4°C. For whole-cell protein extract, a pellet was lysed in 15–300 μl of homogenization buffer: 50 mM Hepes (pH 7.3); 5 mM EDTA; 250 mM NaCl; 5 mM DTT; 0.2% (v/v) NP40 (Sigma Aldrich); and 1% (v/v) Complete Protease Inhibitors Cocktail (Sigma Aldrich). The resuspended pellet was left on ice for 5 min, vortexed, and then centrifuged at 16000 × g for 15 min at 4°C, and extracted proteins remain in the supernatant. Protein concentration was analyzed with the Bradford method (BioRad Protein Assay, BioRad) and kept at -20°C until western blot analysis [16]. For all proteins, 20 μg of whole-cell extracts were resolved by SDS-PAGE on 10% gels. After electrophoresis, proteins were transferred into PVDF membranes (Hybond-P, Amersham Biosciences) using a Novex apparatus (Novel Experimental Technology). Membranes were probed with: 1:1000 anti-cyclin E1 and D1 (Abcam), and 1:10000 anti-cyclophillin A (Abcam). Immunoreactive bands were developed with the ECL Prime system (Amersham Biosciences) and visualized using a digital imaging system (I.Q. LAS 4000 MINI GOLD, GE HealthcareQ9).

### RNA isolation and qPCR

RNA was extracted from PC12 cells as previously described [24]. The reverse transcription (RT) reaction was performed immediately after the mRNA isolation using SuperScript II RNase-H reverse transcriptase (Invitrogen). Real time PCR analysis was carried out in an ABI Prism 7500 Sequence Detection System (Applied Biosystems, Warrington, UK) using SYBR Green PCR Master Mix (Applied Biosystems) and the thermocycler conditions recommended by the manufacturer. Amplification of cyclophillin A DNA was used to normalize for differences in amounts of input DNA between samples. Primers were designed using the Primer Express 2.0 software (Applied Biosystems) and their sequence is indicated in Table 1. Melting curve analysis showed a single sharp peak with the expected Tm for all samples.

**Table 1:** Primer sequences used for the validation by qPCR amplification of different genes.

Gene	Primer Forward	Primer Reverse
**CADM1**	S: 5’-CCAGCAGTTCACGGCCT A-3’	AS: 5’-CGAGAATCTGAGAGATCCTCACTGT-3’
**CDC14B**	S: 5’-GCGGTGCATTGCAAAGCT-3’	AS: 5’-TGCTCTCGGCTGCTGTCAT-3’
**CDH22**	S: 5’-ACAGTGACCATCGTGGTTACTGA-3’	AS: 5’-TCCTGTATGCTGAACTGGTACATCTT-3’
**CXCL9**	S: 5’-AATCCCTCAAAGACCTCAAACAGT-3’	AS: 5’-TCTTCAGTGTAGCGATGATTTCAGT-3’
**CXCL10**	S: 5’-CTGTCCGCATGTTGAGATCATT-3’	AS: 5’-ATGGCCTCAGATTCCGGATT-3’
**IGFBP5**	S: 5’-AGAAAGCAGTGCAAGCCTTCTC-3’	AS: 5’-GGCAGCTTCATCCCATACTTGT-3’
**IL6R**	S: 5’-CCCAGAGGAGCCCAAGCT-3’	AS: 5’-GCTTGGATGCCACTCACAAA-3’
**JUN**	S: 5’-TGATCATCCAGTCCAGCAATG-3’	AS: 5’-TGCTCGTCGGTCACGTTCT-3’
**JUNB**	S: 5’-GACACAGGCGCATCTCTGAAG-3’	AS: 5’-GATCACGCCGTTGCTGTTG-3’
**MMP19**	S: 5’-CGTGGAATCAGTCTTGGATGTTC-3’	AS: 5’-CAAGGTGACATTAGCTGAGGATGA-3’
**NFKB2**	S: 5’-GACGTTCATAAACAGTATGCCATTG-3’	AS: 5’-ACACCGTTACAGGCCTCTCAA-3’
**S100A11**	S: 5’- GCCATCCCAAATGGAGCAT-3’	AS: 5’-CTCAGGTCCTCCTTTGTCAAGTAGT-3’
**S100A10**	S: 5’-GCCATCCCAAATGGAGCAT-3’	AS: 5’-CTCAGGTCCTCCTTTGTCAAGTAGT-3’
**SCRT**	S: 5’-CCAGTGGCACCTTCAAGAGTTC-3’	AS: 5’-TTGCTCAGTGCTAGCCTTGGT-3’
**USP11**	S: 5’-TGGAGACCCGAAACAAAGATG-3’	AS: 5’-CAGATTGGTAAGGCCACAGATG-3’
**ZEB2**	S: 5’-GAAAGCTTCCCAGGTCCTATCC-3’	AS: 5’-GGACCGCCTTGATCTCTTCA-3’
**AQP3**	S: 5’-TCTGGACACTTGGATATGGTCAA-3’	AS: 5’-CAACAATGGCCAGCACACA-3’
**CICLO**	S: 5’-GCACTGGTGGCAAGTCCAT-3’	AS: 5’-GCCAGGACCTGTATGCTTCAG-3’

### Hydrogen peroxide permeability

For this assay cells were transfected with the expression vector pHyPer-Cyto (Evrogen, Moscow, Russia), which codes for a cytoplasmic fluorescence protein (HyPer) capable of detecting intracellular hydrogen peroxide (H_2_O_2_). As indicated by the manufacturer, HyPer demonstrates submicromolar affinity to hydrogen peroxide and is insensitive to other oxidants tested. In the absence of H_2_O_2_, HyPer has two excitation peaks with maxima at 420 nm and 500 nm, and one emission peak with maximum at 516 nm. Upon exposure to H_2_O_2_, the excitation peak at 420 nm decreases proportionally to the increase in the peak at 500 nm, allowing ratiometric measurement of H_2_O_2_.levels. Entrance of H_2_O_2_ into cells (PC12-wt, PC12-AQP3, PC12-AQP1 and PC12-G6PD as control), was measured by monitoring the changes in fluorescence of HyPer after cell exposure to H_2_O_2_. For the experiment, 32 h after pHyPer-Cyto transfection, cells were harvest, washed and resuspended in FACS, and fluorescence changes were monitored with a BD LSR Fortessa flow cytometer (BD Biosciences) using a 488 nm laser and a bandpass emission filter at 530/30. First, HyPer-expressing cells were selected by their basal fluorescence and at that time, their fluorescence was monitored during 2 min before H_2_O_2_ addition. Then, H_2_O_2_ was added to the cell suspension at final concentration of 10 μM, and fluorescence changes of a cell population (~1000 cells) were monitored every 2 min for 16 min. For quantification analysis the basal fluorescence was normalized to 1.

### Statistical analysis

All statistical analyses were conducted using the Statistical Package for the Social Sciences (SPSS 18.0). Data are expressed as mean ± standard error of the mean from at least four different experiments. The statistical significance for normally distributed data was estimated using the Student's t-test for two-group data sets and one-way ANOVA followed by Bonferroni's post-hoc test to compare more than two groups. Data with a non-normal distribution were analyzed using Mann-Whitney U or Kruskal-Wallis H tests for two or for more than two groups, respectively. Values of p ≤ 0.05 were considered significant.

## Results and Discussion

A pro-proliferative action of AQPs in cells that overexpress these proteins have been described in previous works [17,19]. In the present study we performed specific experiments to understand in more detail how expression of AQP3 can modify the cell proliferation process.

**Fig 1:**
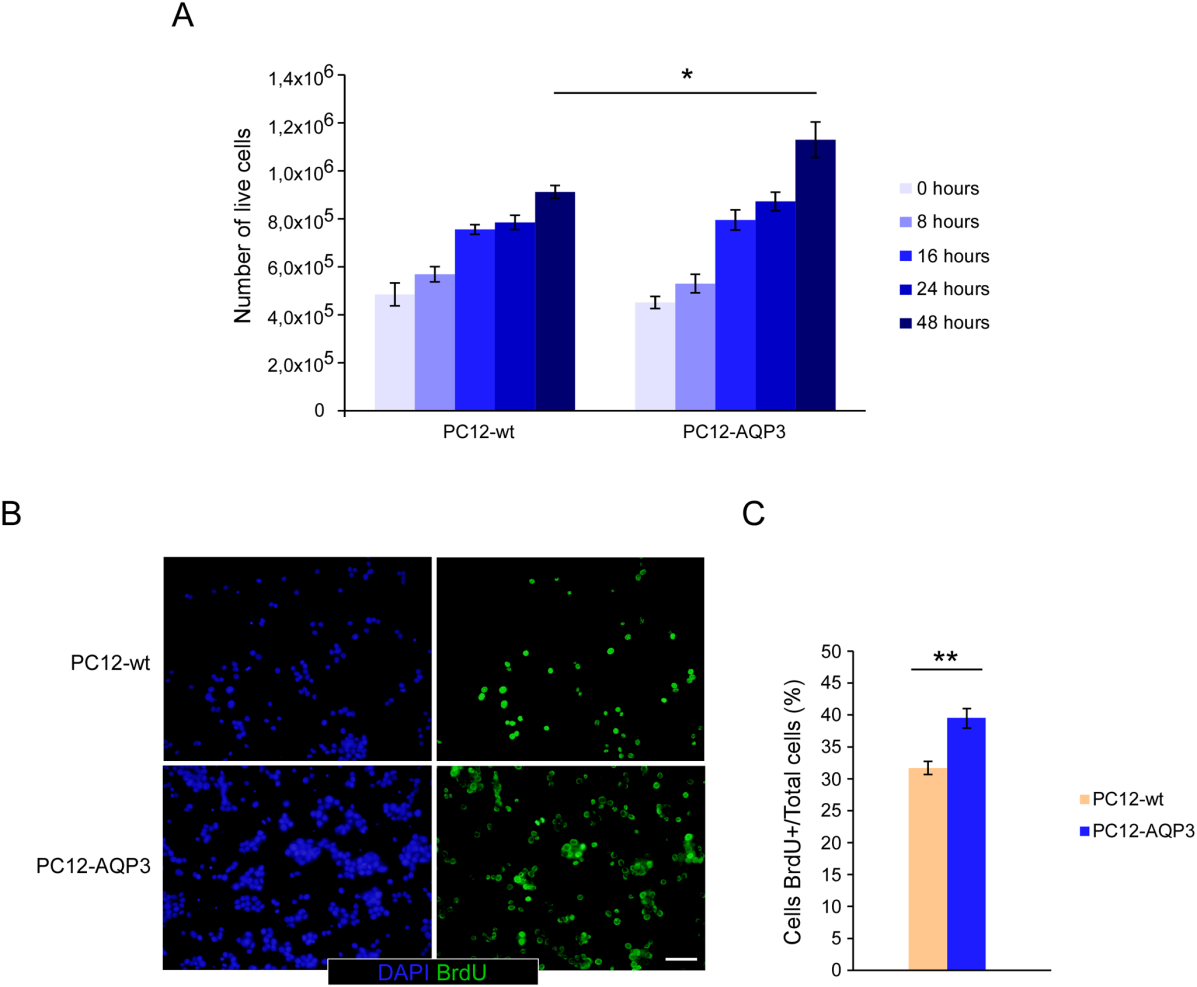
Analysis of cell proliferation rates in cultured cells. (A) Proliferation was assessed by hemocytometer counting of cells not stained with trypan blue at 8, 16, 24 and 48 h after seedtime. Significant differences (* p ≤ 0.05) among both cell lines were observed after 48 h of culture. (B) Analysis of new born cells was performed after BrdU incorporation. Cells were incubated for 8 h with 10 μM BrdU and inmunocytofluorescence microscopy images analyzed for counting of fluorescence cells. An example of these photographs is shown here. (C) Percentage of BrdU+ cells for wt or AQP3 overexpressing cells are shown. Bars are mean ± SEM from 4–7 experiments.

**Fig 2:**
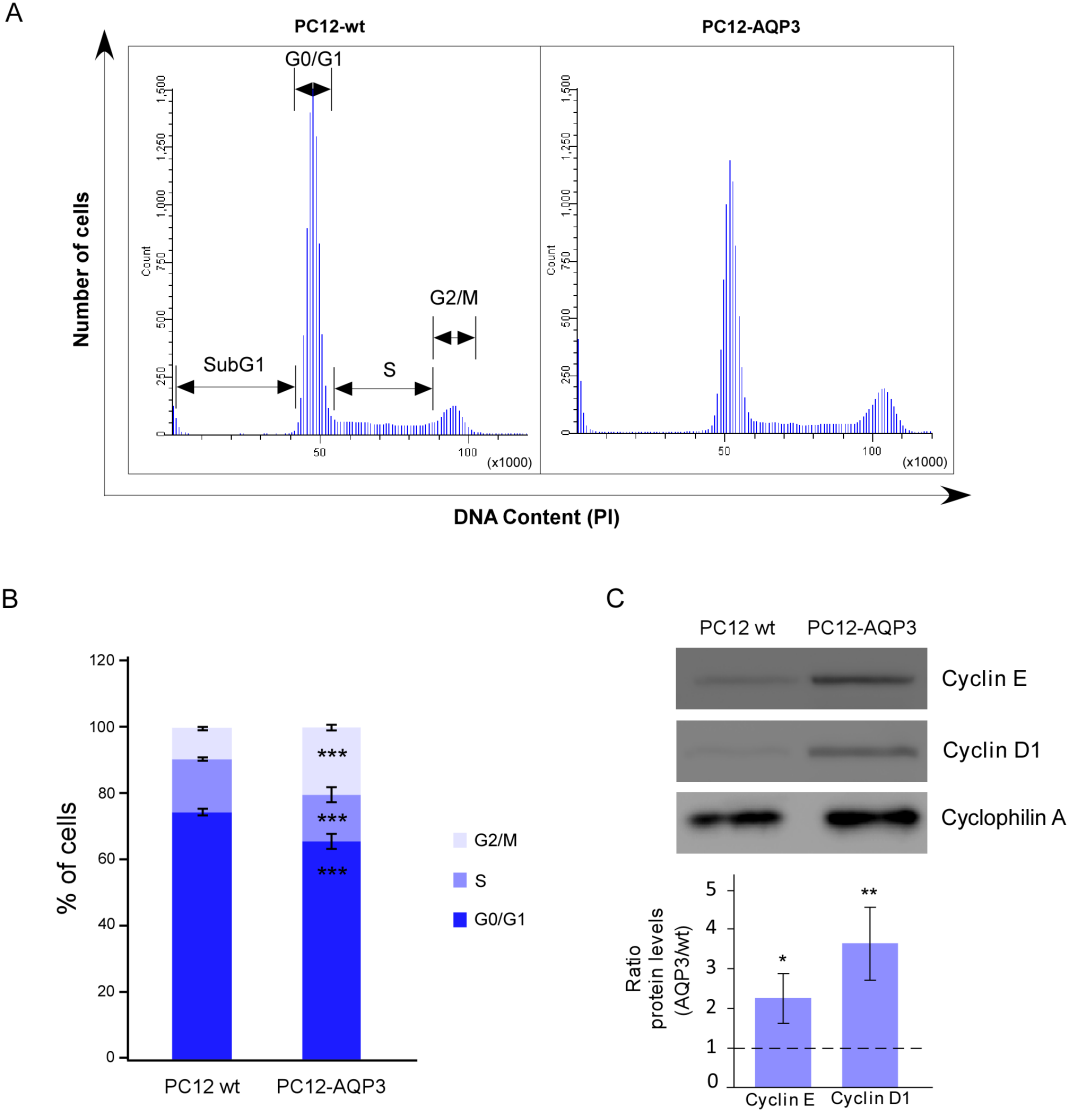
Analysis by flow cytometry of cell cycle profiles. (A). Cell cycle profiles showing the distribution of cells in the different phases of the cell cycle are presented. (B) Summary of the % of cells in each phase of the cycle. Significant differences (*** p ≤ 0.001) are indicated. (C) Western blot analysis of levels for cyclin E and D1 in each cell type (upper panel) and summary of the quantification analysis (bottom panel). The dash line at ratio of 1 indicates levels of cyclins in the wt cells. Bars are mean ± SEM from N experiments where N = 9 in (B) and N = 4 in (C).

We start comparing the proliferation rate of cells that overexpress AQP3 with that of wild type PC12 cells and observed after cell counting and BrdU incorporation measurements (Fig 1) around 20% higher proliferation in cells that overexpress AQP3. A faster progression ramp of growth can be appreciated in AQP3 overexpressing cells by just looking the increment in number of cells over time for each type of cells separately. Moreover, comparative cell counting analysis revealed significant differences only after 48h of seeding (Fig 1A, p≤0.05), a time enough to allow complete cell division; while for BrdU incorporation, 8h of exposure were sufficient to see differences in the measurement of new DNA synthesis (p≤0.01). Thus, these data confirm that cells that overexpress AQP3 have a higher rate of DNA synthesis and grow faster than cells with no expression of this protein. In addition, we investigated whether the overexpression of AQP3 affects the distribution of cells in the different cell cycle phases (Fig 2A). Hence, consistently with a higher proliferative rate, we observed an increase in the percentage of cells in the most proliferative phases, S and G2/M (p≤0.001), in asynchronized cells that overexpress AQP3 (Fig 2B), compared to control cells. At the same time, cells that overexpress AQP3 showed significantly fewer cells in the G0/G1 (Fig 2B, p≤0.001) phase of the cell cycle. These results concurrently with the larger total number of cells described before indicate a more proliferative phenotype; and the possibility for cells being in a replicative senescence state was rule out after observing extremely low number of cells positive for the Beta-Galactosidase assay (data not shown). Furthermore, western blot analysis of levels of cyclins D1 and E1, two key cell cycle regulatory proteins crucial for phase transition during cell cycle progression, showed markedly high levels of both proteins (Fig 2C) in the AQP3-overexpression cell line, contrasting with the low levels detected in control cells. The larger expression of cyclins might well contribute to explain the greater proliferative capacity of AQP3-overexpressing cells and resemble similar findings described in cells with overexpression of AQP1 [23].

**Fig 3:**
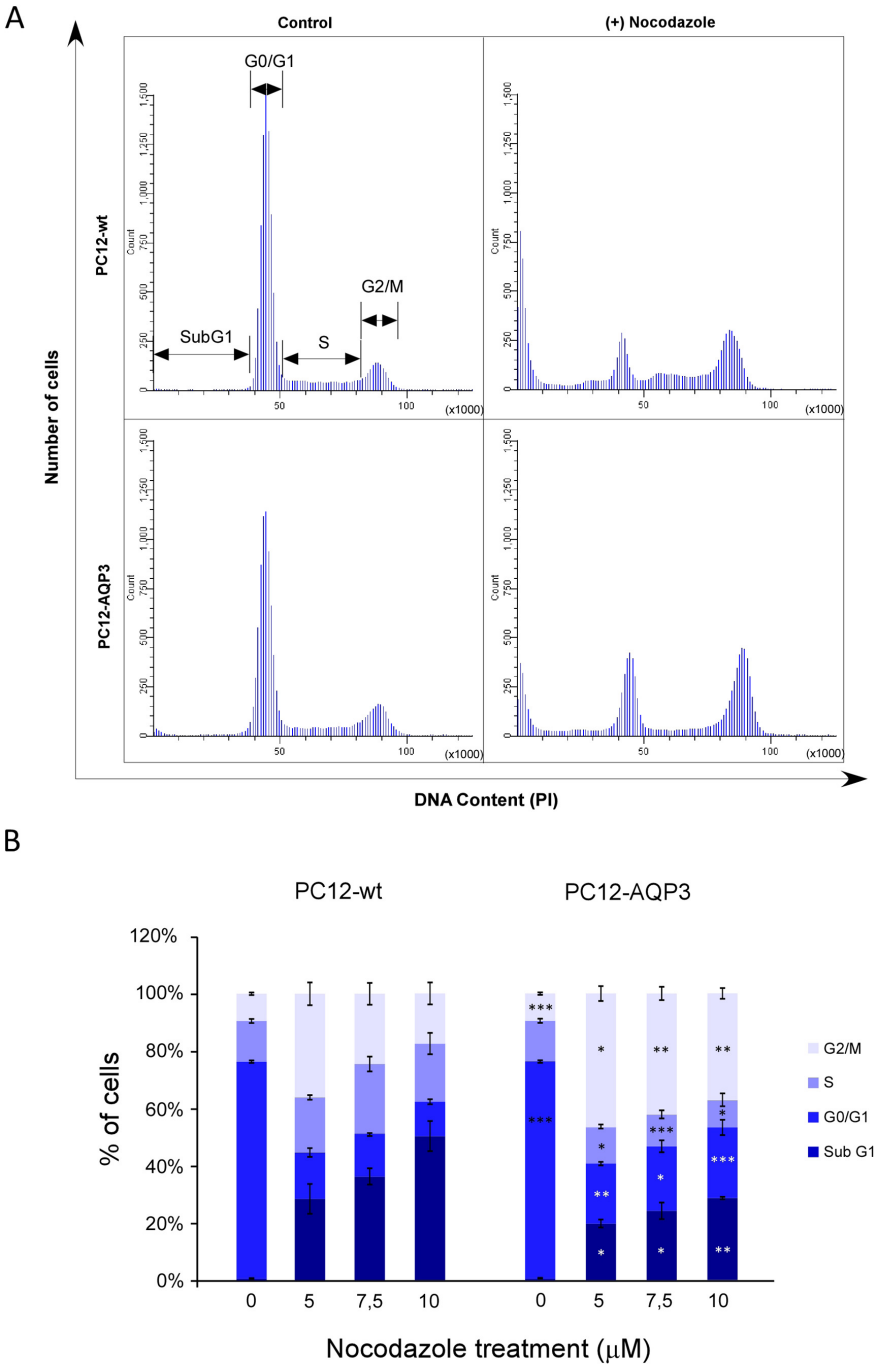
Cell cycle analysis after nocodazole treatment. **(A)** The distribution of cells in the different phases of the cell cycle was analyzed in absence (control) or presence of nocodazole in both cell types, and a representative example of the cell cycle pattern after treatment with 7.5 μM of nocodazole is shown. (B) Summary of percentage of cells in each phase of the cell cycle with different concentrations (5, 7.5 and 10 μM, 24 h) of the drug. Significant differences are indicated as follows: * p ≤ 0.05, ** p ≤ 0.01 and *** p ≤ 0.001. Bars are mean ± SEM from 4–6 experiments.

**Fig 4:**
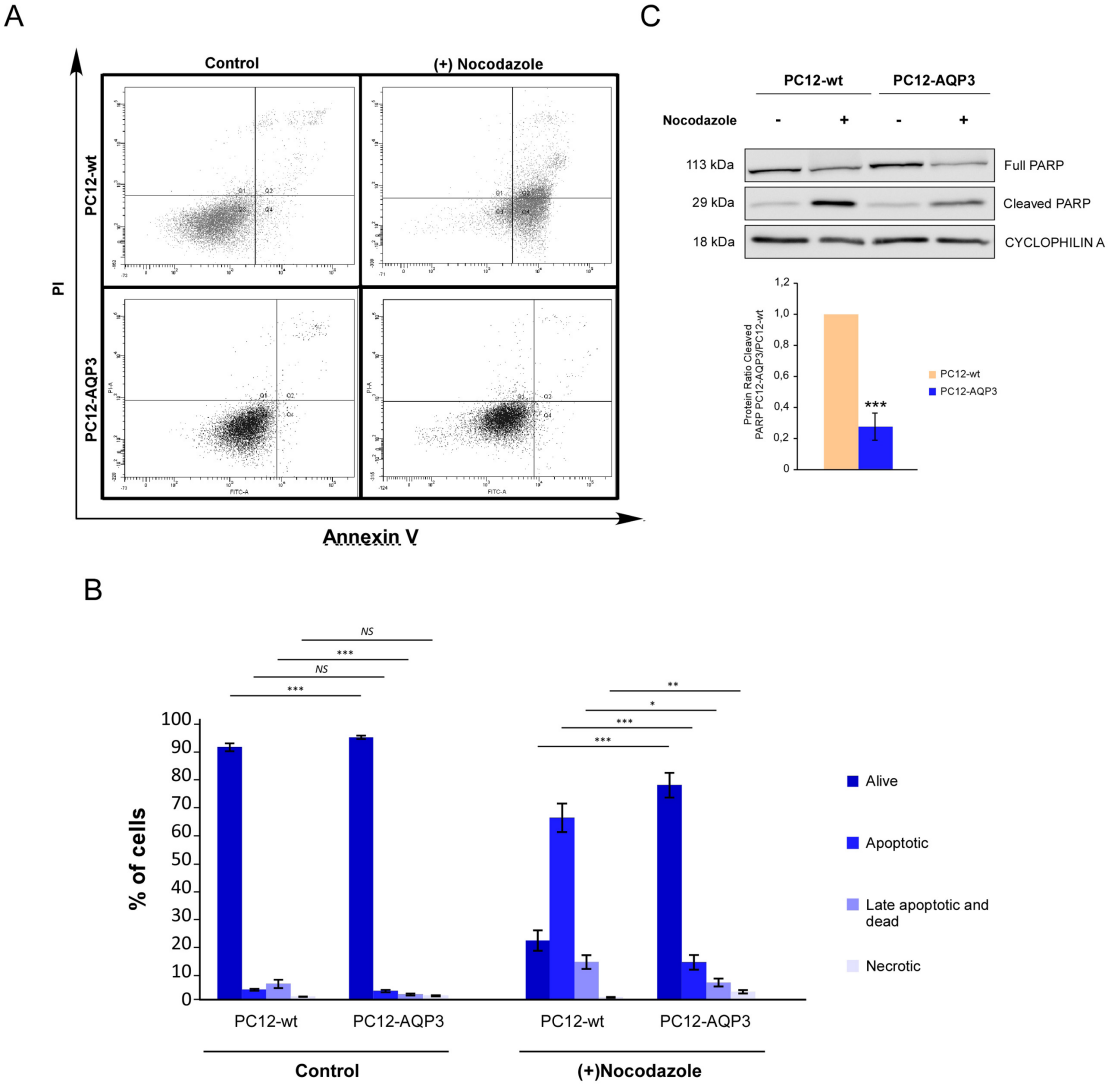
Induction of cell apoptosis by nocodazole treatment. **(A)** Flow cytometry dot plot analysis of annexin V/PI-stained cells to evaluate the induction of apoptosis by nocodazole (7.5μM, 24 h). (B) Summary of the percentage of cells found in each stage: annexin V^-^/PI^-^ (alive), annexin V^+^/PI^-^ (apoptotic), annexin V^+^/PI^+^ (late apoptotic or dead) and annexin V^-^/PI^+^ (necrotic). (C) Analysis by Western blot of levels of intact PARP protein (full) and cleaved PARP (upper panel) and summary of quantification analysis (bottom panel). Error bars are mean ± SEM from N experiments where N = 3 in (B) and N = 4 in (C). Significant differences are indicated as follows: *p ≤ 0.05, ** p ≤ 0.01 and *** p ≤ 0.001.

To further explore how overexpression of AQP3 modifies the cell cycle, this was also studied after nocodazole treatment to synchronize cells in the mitotic prophase. The analysis (Fig 3) showed that cells with overexpression of AQP3 are more resistant to nocodazole effects (Fig 3A). Moreover, both type of cells presented an additional peak in the cell cycle profile corresponding to a new population of cells called SubG1, which corresponds to dead cells (Fig 3A and 3B). Interestingly, the percentage of cells in the SubG1 phase was considerably smaller in the AQP3 overexpressing cells than in control, suggesting that overexpression of AQP3 confers higher resistance to cell death induction by nocodazole treatment. Afterward to specifically explore that cells react differently in response to apoptosis induced by nocodazole [25] we measured staining of cells with annexin V, a specific cell surface marker for apoptosis, in the presence of propidium iodide (PI), and confirmed in both cell types that the number of apoptotic (annexin V^+^) and late-apoptotic (annexin V^+^ plus PI^+^) cells increase after nocodazole treatment (Fig 4A and 4B), but the increment was significantly smaller in AQP3 overexpressing cells than in control (Fig 4B, p≤0.001). Specifically, the percentage of apoptotic cells in both type of cells rise from around 5% in the absence of drug to roughly 15% in AQP3 overexpressing cells and 70% in control cells, after 24h of treatment with 7,5 μM nocodazole (Fig 4B). Western blot analysis of PARP protein levels, another indicator of the cell apoptosis process additionally confirmed reduced induction of apoptosis. Results (Fig 4C) demonstrated a significantly lower level of cleaved PARP after nocodazole treatment in PC12-AQP3 than in PC12-wt. This confirms the idea that overexpression of AQP3 endows cells with considerable resistance to apoptosis induced by nocodazole. Although the mechanism is unknown at this time, similar results have been described with tumor cell lines that became resistant to drugs used for synchronization experiments [26–28].

**Fig 5:**
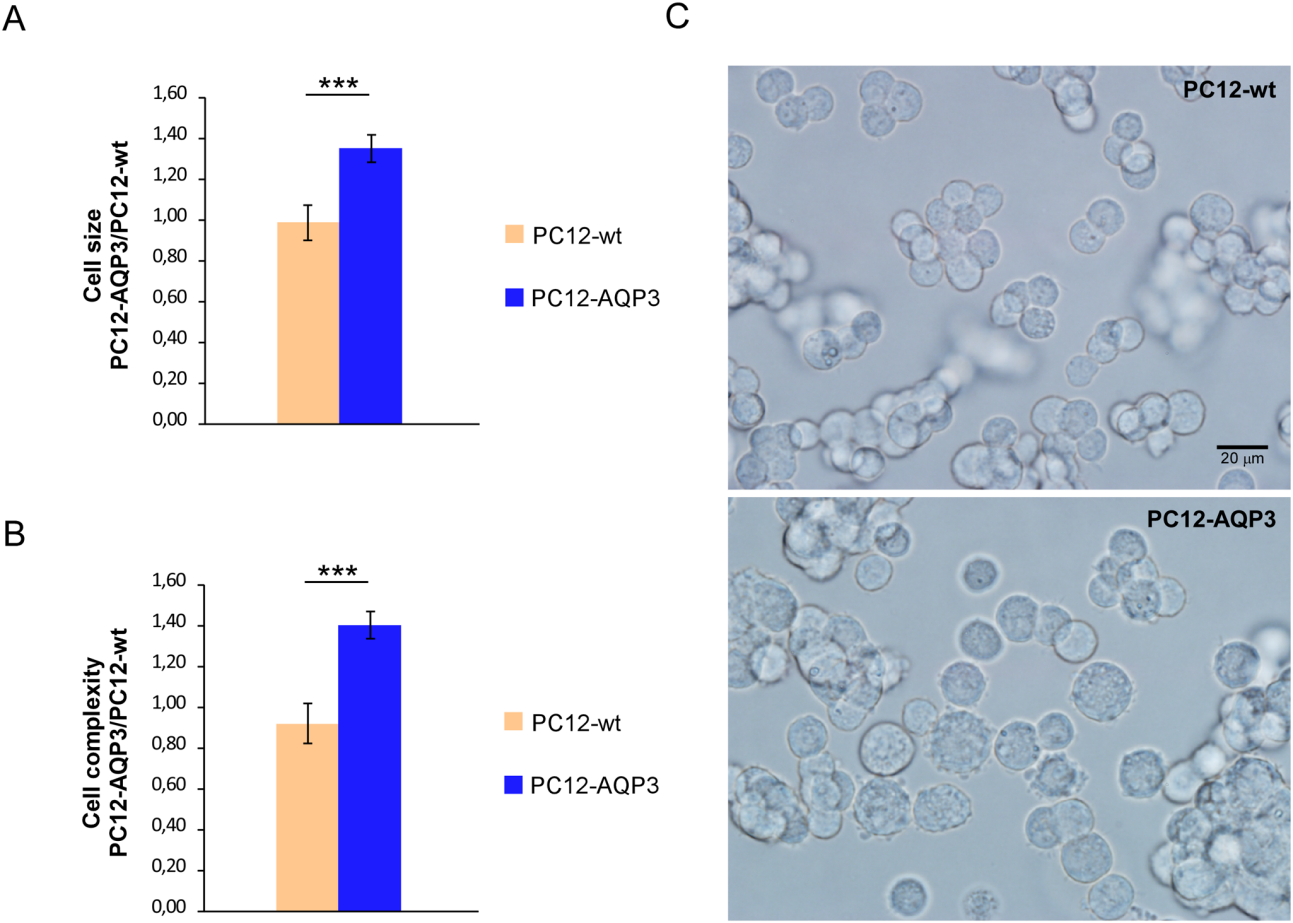
Evaluation of relative cell size and complexity. **(A)** Summary of relative cell size evaluated by flow cytometry using the FSC parameter. (B). Summary of the relative cell complexity evaluated by flow cytometry using the SSC parameter. (C) Light microscope images of PC12-Wt and PC12-AQP3 cells in suspension to show cell morphology. Bars in panel A and B are mean ± SEM from N experiments where N = 8 in (A) and N = 8 in (B). Significant differences are indicated with *** p ≤ 0.001.

As apoptosis is a process known to occur with cell volume reduction [21,29], the constitutive larger volume observed in cells that overexpress AQP3 (Fig 5A and 5C) was likewise in line with the higher resistance to apoptosis observed in cells that overexpress AQPs. Several authors have described the involvement of some AQPs in cell cycle progression and cell death by apoptosis. Thus, expression of AQP2 in collecting duct cells has been associated with acceleration of cell proliferation *per se*, by increasing the rate of cell cycle progression in these renal cells [20,21], and with the rapid regulatory volume decrease that allows these cells to enter into apoptosis [30]. Additionally, since apoptosis involves a reduction in cell volume [21,29], we might speculate that the constitutive larger volume observed in PC12-AQP3 cells could antagonize the apoptotic process. In previous work apoptosis facilitation by AQP1 expression has been indicated before and associated mainly due to its water permeability [31], however the present work and others very recently [23] start to claim that overexpression of AQPs modify cell cycle and proliferation by altering in a stable way the expression of important genes related with cell proliferation and apoptosis resistance.

On the other hand the larger cell complexity (Fig 5B), with a normal cell morphology (Fig 5C), observed in AQP3 overexpressing cells, also fit well with the already described larger proliferative phenotype. Increment in DNA synthesis together with other constitutive elements for cell division would be needed to support a higher cell proliferation rate [32] and would explain the larger value for the complexity parameter.

**Fig 6:**
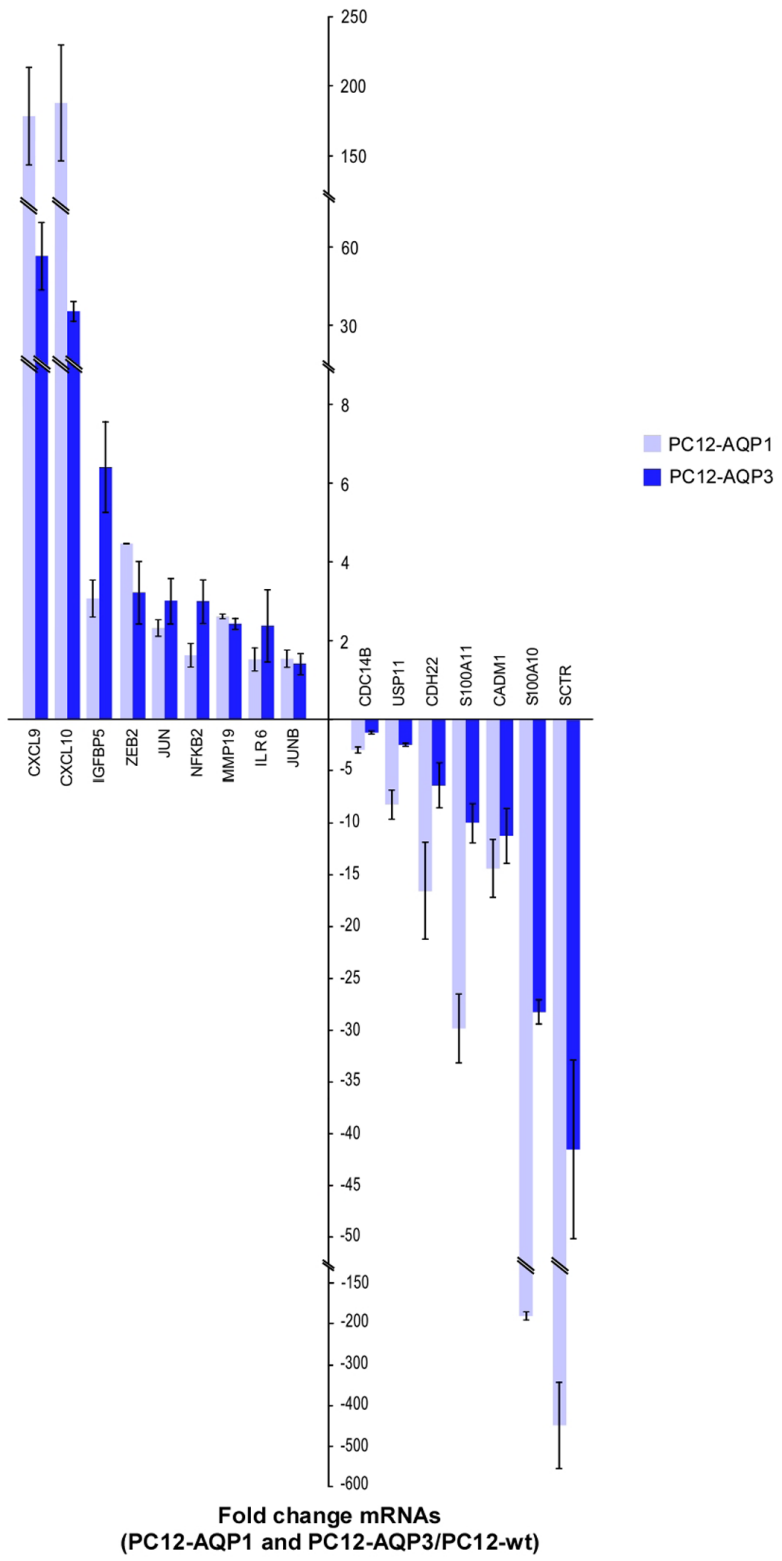
Analysis of gene expression levels. Validation by qPCR analysis of altered expression of genes related with proliferation that were selected based in previous results [23]. In dark blue are shown results obtained in AQP3 overexpression cells (PC12-AQP3) and in light blue are represented results obtained in AQP1 overexpression cells (PC12-AQP1). Bars are mean ± SEM, from N = 3. Analyzed genes were: CXC10: chemokine (C-X-C motif) ligand 10; CXCL9: chemokine (C-X-C motif) ligand 9; ZEB2: zinc finger E-box binding homeobox 2; IGFBP5: Insulin-Like Growth Factor Binding Protein 5; MMP19: matrix metalloproteinase 19; JUN: jun proto-oncogen; NFKB2: subunits of the transcription factor complex nuclear factor-kappa-B (p49/p100); JUNB: jun B proto-oncogen; ILR6: Interleukin 6 Receptor; CDC14B: cell division cycle 14B; USP11: ubiquitin specific peptidase 11; CADM1: Cell adhesion molecula 1; CDH22: cadherin 22, type 2; S100A11: S100 calcium binding protein A11; SCTR: Secretin receptor. Levels of AQP3 mRNA expression evaluated y qRT-PCR in PC12-AQP3 were of more than 1000 fold.

Furthermore, since similar results to those described here were previously observed in a cell line that overexpressed AQP1, and a microarray analysis performed in these cells revealed clear changes in the expression of genes associated with cell proliferation, morphology or cell-movement related functions [23] we decided to explore whether some of those genes would have an altered expression in AQP3 overexpressing cells as well. With qPCR we checked the transcript levels of mRNA for 16 genes and confirmed that expression of all those genes was changed in a similar pattern in both AQP1- and AQP3-overexpression clones (Fig 6). Huge overexpression of two cytokines, CXCL9 and CXCL10, with important pro-tumor roles was detected, and up-regulation of expression of some transcription factors important in cell proliferation (ZEB2, JUN, JUNB and NFKB2), and three other genes (IGFBP5, MMP19, and ILR6) associated to proliferation, cell migration, growth and differentiation were found also overexpressed. On the contrary, down regulation of several other genes (CDC14B, USP11, CADM1, CDH22, S100A10 and S100A11) most of them implicated in regulation of cell cycle progression or in some cases considered as cell tumor suppressors, were similarly reduced in both cell types. Tremendous repression of SCTR (secretin receptor) expression was observed. Curiously, this gene, involved in pancreas cancer [33,34], play also significant role in the regulation by secretin of AQP1 expression in cholangiocytes plasma membrane [35–37]; which may explain the strong down-regulation of SCTR observed in a cell with high overexpression of aquaporins. Altogether the above results strongly support the notion that AQP3 overexpression accelerates the cell proliferation because affects the expression of key proteins for the cell cycle progression and contributes to cell volume changes needed for this process.

**Fig 7:**
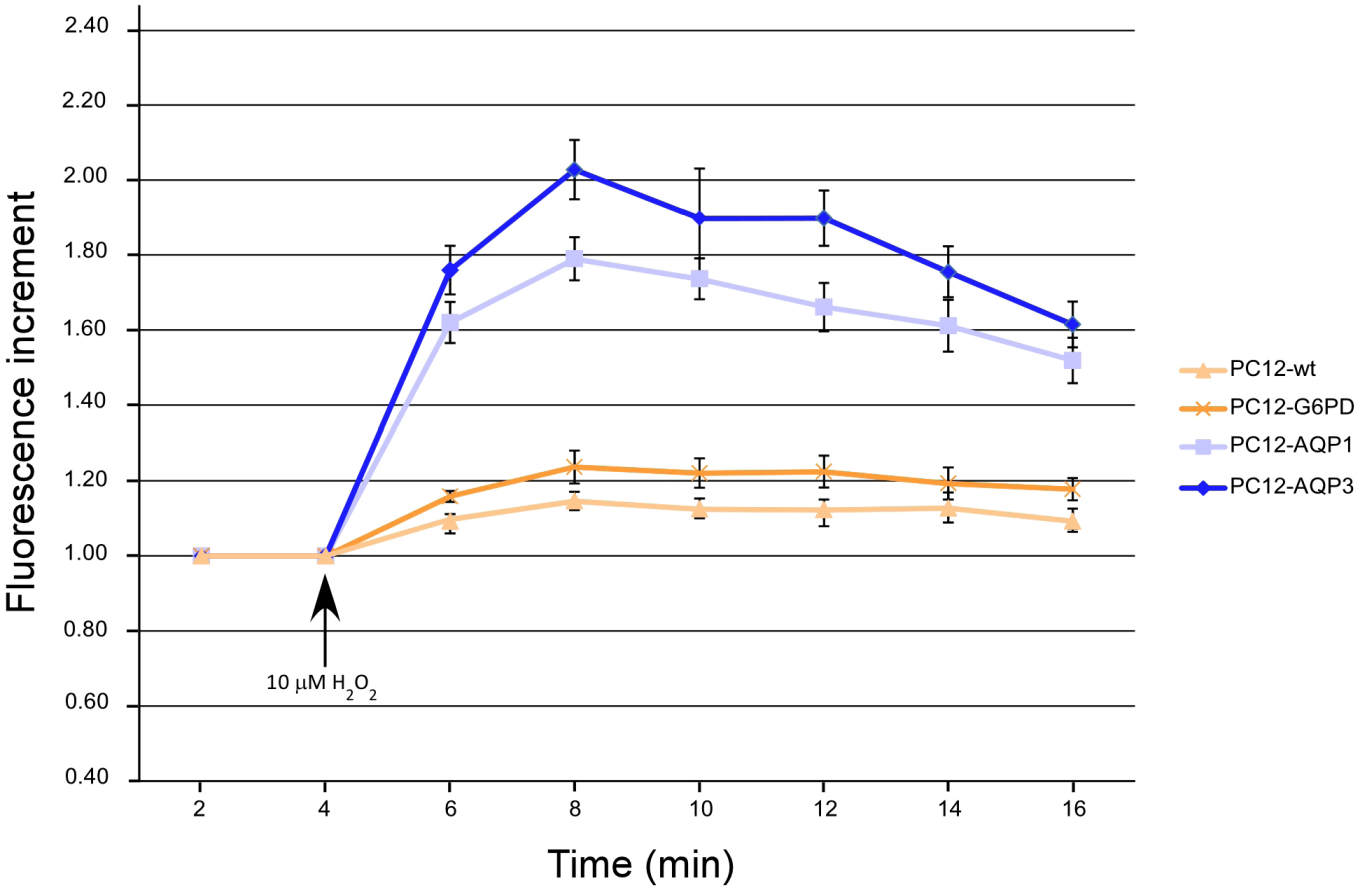
AQPs mediate uptake of H_2_O_2_ in cells. Analysis of hydrogen peroxide uptake in cells expressing the HyPer protein. Time course of changes in the HyPer fluorescence upon treatment of cells with 10 μM H_2_O_2_ were recorded by flow cytometry. At each time point the average of the mean fluorescence signal obtained from ~1000 cells during a 2 min gap interval are shown. Error bars are SEM (N = 5).

In an attempt to further accumulate additional and independent evidences supporting a role for AQP1 and AQP3 in the cell proliferation process, we finish the present study exploring in these AQP-overexpression clones the permeability to hydrogen peroxide. We measured entrance of H_2_O_2_ into cells by monitoring the changes in fluorescence of the HyPer protein after exposure to hydrogen peroxide, but instead of using fluorescence microscopy as used previously [38–40], we recorded HyPer fluorescence changes by using a methodological approach of flow cytometry described here for the first time. The strength of our flow cytometer method, apart from its high sensibility to detect fluorescence changes, relay on the large number of cells analyzed at each time point during data acquisition. The results presented here (Fig 7) showed that both rat AQPs, allowed entrance of H_2_O_2_ to the cells. As others [38–41], we also postulated that entrance of H_2_O_2_ in mammalian systems by AQPs might interfere intracellular signaling, amplifying cascades that depend on ROS, or increase the phosphorylation status of a cell (AKT/protein kinase B) and thus favor proliferation cascades.

Summarizing the main findings of this work into a conclusive paragraph we believe we have demonstrated that AQP3 overexpression modifies the cell cycle pattern increasing the number of cells, upregulating levels of cyclin D1 and E and enhancing DNA syntesis rate. A significantly reduction of the cell death by apoptosis after nocodazole treatment was also observed in AQP3 overexpressing cells. Finally, the large cell volume and complexity, as well as enhanced hydrogen peroxide permeability and altered expression of proteins necessary for progression of the cell cycle were all findings that contribute to explain the more proliferative phenotype of cells that overexpress AQP3.
